# Anastomotic leak management after a low anterior resection leading to recurrent abdominal compartment syndrome: a case report and review of the literature

**DOI:** 10.1186/1752-1947-3-125

**Published:** 2009-11-14

**Authors:** Kostas Toutouzas, Eleftheria S Kleidi, Panagiotis G Drimousis, Margarita Balla, Metaxia N Papanikolaou, Andreas Larentzakis, Dimitrios Theodorou, Stylianos Katsaragakis

**Affiliations:** 1 1st Department of Propaedeutic Surgery, Surgical Intensive Care Unit, University of Athens, Athens Medical School, Hippocration Hospital, Athens, Greece; 2 Intensive Care Unit, Hippocration Hospital, Athens, Greece

## Abstract

**Introduction:**

Low anterior resection is usually the procedure of choice for rectal cancer, but a series of complications often accompany this procedure. This case report describes successful management of an intricate anastomotic leak after a low anterior resection.

**Case presentation:**

A 66-year-old Caucasian man was admitted to our hospital and diagnosed with a low rectal adenocarcinoma. He underwent a low anterior resection but subsequently developed fecal peritonitis due to an anastomotic leak. He was operated on again but developed abdominal compartment syndrome, multi-organ failure and sepsis. He was aggressively treated in the intensive care unit and in the operating room. Overall, the patient underwent four laparotomies and stayed in the intensive care unit for 75 days. He was discharged after 3 months of hospitalization.

**Conclusion:**

Abdominal compartment syndrome may present as a devastating complication of damage control laparotomy. Prompt recognition and goal-directed management are the cornerstones of treatment.

## Introduction

Low anterior resection (LAR) of the rectum is the best curative procedure for mid and distal rectal carcinomas. Anastomotic leaks occur in approximately 3 to 15% of patients undergoing colorectal surgery and can lead to significant morbidity and mortality [1-3]. The most important risk factor in the development of a leak is the level of the anastomosis. Low rectal anastomoses have a much higher leak rate compared with intraperitoneal colonic ones [1-3]. Anastomotic dehiscence can appear as a clinically silent radiological finding or cause severe sepsis associated with abscesses or peritonitis. Its mortality ranges between 10 and 50% [2].

In cases of diffuse peritonitis, immediate exploratory laparotomy is needed. The options for surgical treatment include resection of the anastomosis with proximal diversion, single drainage of the anastomosis and proximal diversion with a loop ostomy [3]. Although reported to significantly increase mortality, these procedures have possible serious complications. To our knowledge, there is no preferred management of such complications in the available literature. The presence of peritonitis, as well as damage control laparotomy, constitutes a risk factor for the development of abdominal compartment syndrome (ACS). ACS can cause further deterioration in the patient's condition. Appropriate intra-operative measures should always be taken to prevent its occurrence.

We present a case of successful anastomotic leak management after a low anterior resection, in a man with adenocarcinoma of the lower rectum. We focus on the type of operative procedures performed in the presence of recurrent ACS.

## Case presentation

A 66-year-old Caucasian man was admitted to our surgical department with the diagnosis of a rectal cancer 8 cm from the anal verge. He had a colonoscopy due to the presence of blood in his stools for a month prior to his admission. Endoscopy revealed a polypoid lesion of the rectum and the pathology diagnosed an adenocarcinoma.

From his medical history, we ascertained that he had pulmonary tuberculosis at the age of 25 years, cholocystectomy at the age of 61 and that he currently had ischemic heart disease. The patient had no history of tobacco smoking and no allergies. Laboratory tests were normal. Preoperative staging was negative on abdominal and chest computed tomography scans. The patient did not receive neoadjuvant therapy.

A laparoscopic low anterior resection was performed with an end-to-end anastomosis. The operation was converted to an open laparotomy due to technical problems in the completion of the anastomosis and the intraoperative observation of oozing from the presacral fascia. Microscopic examination of the specimen revealed a moderately differentiated adenocarcinoma of the rectum with adequate resection margins and no metastases in the 16 resected lymph nodes. By World Health Organisation classification, this was a T3N0 M0 tumor.

The immediate postoperative course was uneventful. On the fourth postoperative day, the patient developed abdominal pain and dyspnea. Physical examination revealed tachycardia (130 beats per minute), hypertension (systolic blood pressure of 210 mmHg) and fever (38.1°C). The patient had a normal blood count and hypoxia was diagnosed on arterial blood gases (saturation 88%, PO_2 _56 mmHg). He received intramuscular analgesics (pethidine 75 mg four times daily) and intravenous antibiotics (piperacillin-tazobactam 4.5 gm and metronidazole 500 mg three times daily).

The patient underwent an emergent laparotomy on the same day. Anastomotic rupture with fecal peritonitis was diagnosed. Intraperitoneal lavage and a loop ileostomy were performed. Two drain catheters were placed in the pelvis next to the anastomosis and the abdomen was left open for a delayed closure using the Bogota bag technique. Intraoperatively, the patient suffered a cardiac arrest and he was successfully resuscitated. He was transferred to the intensive care unit (ICU) and mechanically ventilated. He was hemodynamically unstable.

On the seventh day, the patient underwent a second laparotomy, and then returned to the ICU. Due to persistent hyperpyrexia, tachycardia and metabolic acidosis unresponsive to supportive measures, the patient was taken back to the operating theatre for a third laparotomy. Exploration of the peritoneal cavity was negative. We performed peritoneal lavage and three drains were placed in the pelvis. Using the Bogota bag technique, the abdominal wall was left open. The patient was transferred to the ICU again.

Five days later, we detected intestinal content in one of the drain catheters - the patient was hemodynamically stable but his condition was not improving. At that time, abdominal hypertension developed (21 mmHg) accompanied by acute renal failure. It was decided that the patient should undergo another exploratory laparotomy some days later. Fecal peritonitis and pseudomembranes were recognized and a peritoneal lavage with warm saline was performed. The anastomosis was taken down and the rectal stump was closed. Omentum was placed in the lesser pelvis. A proximal sigmoidostomy was performed along with reconstruction of the loop ileostomy. Two drains were placed in the pelvis. The abdominal fascia was left open with skin closure. The patient was intubated and transferred back to the ICU.

The patient developed multi-organ dysfunction requiring mechanical ventilation, inotropic support and renal replacement therapy in his 40 days in the ICU. 17 days after the last laparotomy, he was tracheostomized and was transferred to a hospital ward after 75 days in the ICU. He was on a T-piece and receiving enteral feeding through a Levin tube. Multiple broad-spectrum antibiotics were administered. He was discharged from the hospital and transferred to a rehabilitation center after 3 months of supportive therapy. At that time, he had neither clinical nor laboratory signs of infection, his tracheostomy had closed and he had restarted oral food intake.

## Discussion

Anastomotic disruption is perhaps one of the most dreadful complications a patient can have after intestinal surgery. In the last two decades, the widespread adoption of the total mesorectal excision technique for resection of cancer of the middle and distal rectum has produced leakage in more than 10% of cases [4-6]. Independent factors associated with a higher incidence of leak are anastomosis within 5 cm of the anal verge [5,6], male gender [4-6] and nonconstruction of a proximal diversion [4,5]. The level of anastomosis has been shown to be the most important determinant of anastomotic leakage [5,6]. Many anastomotic leaks do not result in complete disruption and fecal peritonitis. The leak can be small or even occult, yet devastating [5]. Mortality after anastomotic leak ranges from 7.5 to 36% and they are the most common cause of death after colorectal surgery [1-3]. Mortality rate after anastomotic leak has been reported to be as high as 50% in some series of cases of low rectal anastomosis.

The consequences of an anastomotic leak range from diffuse peritonitis, intra-abdominal abscess to enterocutaneous fistula. When a patient experiences an anastomotic leak as well as fever, acute abdominal pain and tenderness early in the postoperative period, there is little doubt about the diagnosis. In these cases, urgent repeat laparotomy for peritoneal lavage and fecal diversion is generally indicated. Prolonged ICU stay and death are not uncommon [3]. In the case of diffuse peritonitis, infection control can be attained by various surgical techniques. But in all cases, three tasks must be accomplished: eliminating the prime source of infection, obliterating all contaminated and/or necrotic material, and preventing recurrent sepsis [2].

### Eliminating the prime source of infection

As was the case in our patient, an immediate exploratory laparotomy is imperative for the eradication of the source of infection in generalized peritonitis. Surgical options for anastomotic leak include either fecal diversion with a diverting loop ileostomy or colostomy, or resection of the anastomosis and creation of an end ostomy (Hartmann's procedure). Diversion with a loop ostomy requires less operative stress and allows a convenient restoration of intestinal continuity at a later stage. However, it leaves *in situ *a septic focus, predisposes to bowel stenosis and chronic fistula, and is feasible only if the colon above the anastomosis is viable. The Hartmann's procedure is more radical and effective at controlling the leak, but restoring intestinal continuity at a later time can be challenging and requires a new laparotomy. As a consequence, many end stomas are never reversed [2,3].

Many studies indicate that the Hartmann's procedure should be the procedure of choice for an anastomosis located above the peritoneal reflection. Proximal diversion may, however, be more appropriate in anastomosis situated below the peritoneal reflection. Hartmann's is used as a last resort in cases of major dehiscence (more than 50% of the circumference) or in cases of colon necrosis [2,3].

In the first re-exploration of our patient, the anastomotic dehiscence was minor. Since it was located below the peritoneal reflection (<5 cm from the anal verge), we decided to make a proximal diversion. A loop ileostomy was constructed and the anastomosis was left *in situ*. The anastomosis was not resected (in either the first or the second exploration), due to the absence of macroscopically obvious fecal peritonitis at the time. Apparently, this was not adequate since fecal peritonitis finally redeveloped and the dehiscence in the third re-laparotomy was more than 50% of the anastomotic circumference. The proximal colon appeared ischemic so we proceeded to anastomosis resection and an end sigmoidostomy.

### Obliteration of all infected/necrotic material

In all cases of anastomotic leak, complete dissection is required in order to expose the peritoneal cavity and visualise the anastomosis. Liberal irrigation with copious amounts of warm saline is indicated to reveal the peritoneal surfaces and to remove infected material [2]. Surgical drainage of the anastomosis is required when it is left *in situ *[3].

### Prevention of recurrent sepsis

Successive lavage and drainage of the peritoneal cavity aim to decrease the high mortality in patients with postoperative sepsis [2]. Drains should be placed in the lesser pelvis at the end of the procedure and should be removed only when absolutely indicated [4,5]. Open abdomen management is preferred to primary skin closure in order to reduce the tension of the peritoneal cavity and to avoid the development of ACS and multiple organ failure. Many methods of temporary abdominal closure have been described. The Bogota bag used in this case consists of a simple, safe, inexpensive, tension-free technique that is applied to patients with abdominal sepsis and planned re-explorations. It provides non-desiccating coverage of the viscera and prevents fluid losses and evisceration [2,3].

Although diversion and Hartmann's procedure after a low anterior resection leak are reviewed in the literature, drainage procedures and management are merely reported. In our case report, revised re-operation was indicated due to the patient's physiological signs but conducting an additional peritoneal lavage and the placement of extra drain catheters did not prove adequate in controlling sepsis. The patient developed intra-abdominal hypertension, despite the free-tension closure technique.

### Recurrent abdominal compartment syndrome

Intra-abdominal pressure (IAP) ranges from 5 to 7 mmHg with the upper limit generally accepted by the World Society to be 12 mmHg [7] in healthy individuals. Among the critically ill, IAP is frequently elevated above the patient's normal baseline. Recent abdominal surgery, sepsis, organ failure, need for mechanical ventilation and changes in body position are all associated with elevations in the IAP [8].

Intra-abdominal hypertension (IAH) is defined as a constant increase of the abdominal pressure above 12 mmHg. In our case, the IAP of 21 mmHg is ranked as IAH grade III - out of four grades of IAH classification [7,8]. IAH is a known cause of organ dysfunction in patients after emergent abdominal surgery and trauma [7,9]. Excessive IAH leads to serious pathologic derangements in diverse organ systems, all of which are related to the decreased preload, increased afterload and extrinsic compression, with decreased end-organ oxygen delivery and consumption [7]. The development of IAH during ICU stay has proven to be an independent predictor of mortality [9].

The clinical picture resulting from a sustained IAP above 20 mmHg, which is associated with a new onset of an organ dysfunction and/or failure, is defined as abdominal compartment syndrome (ACS) [8]. As observed in our case, in the setting of IAH, oliguria and elevation of serum creatinine values are identified after acute renal failure. IAH and the ACS cause oliguria in the patient, as the renal injury is still unresponsive to fluid load [7]. Few nonsurgical approaches are available for ACS [9], and surgical decompression is the only definite treatment [7,9]. Decompressive laparotomy and temporary abdominal wall closure until the source of intra-abdominal hypertension is corrected constitute the gold standard for ACS [7].

In our case, the patient developed ACS despite the attempt of the first repeat laparotomy to obviate IAH by maintaining an open abdomen. In such cases, the ACS is referred to as recurrent ACS or ACS of the open abdomen [8-10]. Due to the critical illness of the patients, recurrent ACS is associated with a significant morbidity and mortality of 60% [9,10].

Recurrent ACS is commonly treated by revised surgical decompression but there are hardly any reports on specific operative management choices. In our case, the resection of the septic source, the anastomosis, concomitantly with its isolation from the peritoneal cavity using an omental patch, proper use of drain catheters, and the substitution of the Bogota bag technique with the musculo-fascial separation technique, proved effective in decreasing the intra-abdominal pressure.

The decrease in IAP after decompression does not necessarily reflect an improvement in organ function but many of the adverse effects of IAH are reversible if the IAP is promptly decreased. There may be a positive effect on oxygenation, but the respiratory function of the patient may still remain severely impaired [9]. ICU stay in such cases may be prolonged, and management of sepsis is challenging. Standard therapy includes antibiotics, infection source control and hemodynamic support with fluids and vasoactive medications. Postoperative continued monitoring of intra-abdominal pressure is essential for the recognition of recurrence and is also used to determine the optimal time for abdominal wall closure. Satisfactory systemic oxygenation, euvolemia and amendment of potential coagulopathy are all necessary prerequisites. In our case, postoperative ACS management in ICU resulted in an effective control of sepsis with restoration of all organ dysfunction.

## Conclusion

Although not uncommon, anastomotic leaks after LAR may have a lethal outcome. This can be irrespective of the use of the indicated surgical treatment and the measures taken for the prevention of ACS. IAH in the open abdomen is a rare complication that should not be underestimated. Prompt recognition of IAH and additional decompressive laparotomy is indicated for correcting the septic process. More data need to be reviewed on the treatment of complications of repeat laparotomy after anastomotic leak following low anterior resection.

## Abbreviations

ACS: abdominal compartment syndrome; CT: computer tomography; ICU: intensive care unit; IAP: intra-abdominal pressure; IAH: intra-abdominal hypertension; LAR: low anterior resection.

## Consent

Written informed consent was obtained from the patient for publication of this case report and any accompanying images. A copy of the written consent is available for review by the Editor-in-Chief of this journal.

## Competing interests

The authors declare that they have no competing interests.

## Authors' contributions

TK assisted in the final operation and contributed to postoperative management, manuscript conception and writing. KSE contributed to research, acquisition of data, analysis, drafting and writing of the manuscript. DGP contributed to postoperative management, writing, organizing and critical review of the manuscript. BM contributed to postoperative management, analysis and acquisition of data. PNM contributed to postoperative management, acquisition and interpretation of data. LA contributed to research, drafting, organizing and writing of the manuscript. TD contributed to conception and critically revised the manuscript. KS carried out the final operation and contributed to postoperative management, manuscript conception, acquisition of consent and critical review of the manuscript. All authors read and approved the final manuscript.
